# Case of Rupture of the Bladder: Recovery

**Published:** 1846-12

**Authors:** 


"*Case of Rupture of the Bladder: Recovery.* By WILLIAM CHALDECOTT, Esq., Surgeon, Dorking.—(Read at the Annual Meeting of the South-Eastern Branch of the Provincial Medical and Surgical Association, June 24, 1846.)—The records of surgery exhibit so few instances of recovery after rupture of the urinary bladder, that probably the details of the following case may not be unacceptable to the members of our Association, affording as they do encouragement to both surgeon and patient to hope under circumstances that have generally forbid any other than the worst anticipations.

At 12 o'clock on the night of Tuesday, the 7th of April, Mr. John Philps, a wine merchant in Dorking, a healthy and temperate man, of about fifty, having passed two or three hours at a concert, ran across the street to empty his distended bladder, and the night being dark, he did not see a newly erected post, with the top of which the lower part of his abdomen came in violent contact. He states that he fell, and with great difficulty reached his home, which was about a hundred yards distant.

I saw him about half an hour after the accident. He was faint, and suffering severe pain over the stomach and belly, with desire but no power to pass his urine. I ascertained that none had escaped into his clothes, and my suspicicions as to the nature of the mischief, were confirmed by the circumstance of nothing escaping through a full-sized catheter which was passed easily and completely into the bladder. He was placed in bed, and hot fomentations were used to the belly until re-action took place, with which came increase of pain over the stomach and abdomen. Twenty leeches were also applied, and I now passed a gum catheter, but with the same unsatisfactory result as before, not a drop of urine escaping through it. I wished to have left the instrument in the bladder, but to this the patient strongly objected, and I urged it the less from some apprehension that in his restless movements the point might be forced through the wound in his bladder; the catheter was therefore passed every three or four hours, although up to two o'clock, p. m., fruitlessly.

I apprised the friends of the patient of the nature of the injury, and the extreme danger attending it, and in consequence Mr. Key was sent for. He arrived at six o'clock, which was about eighteen hours after the accident, by which time the symptoms of peritonitis had increased to an alarming degree. The belly was painful, swollen, and tender; the pulse rapid and feeble, and the countenance anxious. Mr. Key passed a catheter, (none having been used for the previous four hours,) and about an ounce of bloody urine came through the instrument. Mr. Key concurred with me in his opinion as to the nature of the injury and the nearly hopeless prospect for the patient.

At ten o'clock I gave him two scruples of liquor opii sedativus, which after a few hours produced some comfortable sleep, and about four hours from the time of Mr. Key's visit I again passed the catheter, and drew off about four ounces of clear urine.—From this time, the pain, swelling, and heat in the stomach and abdomen gradually lessened, and it was evident that the bladder now held, as on each introduction the catheter brought away clear urine.

From this time until the 13th, (that is the sixth day from that on which the accident happened,) all went on well, excepting that a smart attack of gout occurred on the 10th, although the patient had never before suffered one; but on the 13th, from a strong desire to become independent of the catheter, he made straining efforts to pass his water, and he had scarcely passed a tablespoonful, when he felt (to use his own expression,) something give way, and a burning pain all over his stomach and bowels, as if boiling water had been poured over them, and the same symptoms of faintness and distress occurred as when the accident first happened.

I saw him within a few minutes of this re-opening of the wound of his bladder, for I could not doubt but such had been the consequence of his attempts to pass his water. On using the catheter, not more than a teaspoonful came through the tube. He had now again the symptoms of peritonitis, with the addition of incessant sickness. The same plan of treatment was again adopted—viz., fomentations, leeches, and a full opiate, with calomel.

About four hours after, on the introduction of the catheter, the bladder was again found to retain its urine; and although the peritonitis had increased to a severe degree, the pain, tenderness, and sickness, gradually subsided; and by a patient submission to the continued use of the catheter for a fortnight, no more interuption to the patient's amendment occurred, excepting that the gout, which, under the use of colchicum, had nearly disappeared, again became severe, no doubt, from the fresh absorption of urine which this second accident had permitted to escape into the cavity of the peritoneum. But by this time it was presumed that the wound in the bladder had closed with sufficient firmness to allow the patient to yield to the desire to evacuate his urine without any straining efforts.

Two months have now transpired since the commencement of the case, and he feels no other inconvenience from his accident except a dragging sensation over the abdomen, chiefly on the right side, which is much increased when he attempts to lie upon his left side. This no doubt results from adhesions, consequent upon the peritonitis.

*Remarks.*—The manner in which the accident happened, and all the circumstances and symptoms which followed it, were to me conclusive of the nature of the mischief. If anything could suggest a doubt about the rupture of the bladder, it was the very circumstance which makes the case remarkable—namely, the recovery; but to one who watched the case, no such doubt could occur, since it must be recollected that the bladder was known to be full at the time of the accident, no urine escaped into the clothes, nor did any come through the urethra for more than eighteen hours after, and then only about an ounce, mixed with blood. If, therefore, it did not escape into the cavity of the peritoneum, what else could have become of it?

This case may range with many, in proof of what severe injury the peritoneum may sometimes sustain, and the patient yet survive. Indeed out of the evil of peritonitis, which usually renders this accident fatal, came the good of such an effusion of lymph, as no doubt glued the bladder where wounded to the contiguous viscera.

The fact of gout occurring upon the absorption of urine which first escaped into the peritoneum, and its aggravation upon its second extravasation, is interesting as connected with the pathology of that disorder."—*Prov. Med. and Surg. Jour.*

